# Choice of birth place among antenatal clinic attendees in rural mission hospitals in Ebonyi State, South-East Nigeria

**DOI:** 10.1371/journal.pone.0211306

**Published:** 2019-11-05

**Authors:** Leonard O. Ajah, Fidelis A. Onu, Oliver C. Ogbuinya, Monique I. Ajah, Benjamin C. Ozumba, Anthony T. Agbata, Robinson C. Onoh, Kenneth C. Ekwedigwe

**Affiliations:** 1 Department of Obstetrics and Gynaecology, Faculty of Medical Sciences, University of Nigeria, Ituku-Ozalla Campus, Enugu, Nigeria; 2 Department of Obstetrics and Gynaecology, Federal Teaching Hospital, Abakaliki, Nigeria; 3 Institute of Maternal and Childhealth, University of Nigeria, Ituku-Ozalla Campus, Enugu, Nigeria; University of Ghana College of Health Sciences, GHANA

## Abstract

**Background:**

Low utilization of health facilities for delivery by pregnant women poses a public health challenge in Nigeria.

**Aim:**

To determine the factors that influence the choice of birth place among antenatal clinic attendees.

**Methodology:**

This was a cross-sectional study of the eligible antenatal clinic attendees recruited at Mater Misericordiae Hospital, Afikpo and Saint Vincent Hospital, Ndubia in Ebonyi State from February 1, 2016 to June 30, 2016. Analysis was done using EPI Info 7.21 software (CDC Atlanta Georgia).

**Results:**

A total of 397(99.3%) completely filled questionnaires were collated and analysed. Approximately 71% of the health facilities closest to the respondents had maternity services. It took at least 1 hour for 80.9% of the respondents to access health facilities with maternity services. Most (60.2%) of the respondents had at least one antenatal clinic attendance and majority of them did so at public hospitals. Approximately 43.8% of the respondents were delivered by the skilled birth attendants. The respondents’ age and the couple’s educational level, history of antenatal clinic attendance, distance of the health facility and availability of transport fare had a significant effect on delivery by skilled birth attendants. The common determinants of birth place were nearness of the health facilities, familiarity of healthcare providers, improved services, sudden labour onset and cost. Also 61.7% of the respondents chose to deliver in public health facilities due to favourable reasons but this could be hampered by the rudeness of some healthcare providers at such facilities. A significant proportion of private health facilities had unskilled manpower and shortage of drugs.

**Conclusion:**

A greater proportion of women will prefer to deliver in health facilities. However there are barriers to utilization of these facilities hence the need to address such barriers.

## Introduction

According to the United Nations International Children’s Emergency Fund (UNICEF), a woman dies from complications of childbirth every minute.[1] Therefore about 303,000 women die annually from complications of childbirth.[2] A woman in sub-Saharan Africa has 1 in 16 chances of dying in pregnancy or childbirth, compared to 1 in 4,000 risks in developed countries.[1] This makes maternal mortality the health indicator showing the greatest disparity between the developing and developed countries.[3] The 2013 Nigerian Demographic and Health Survey showed that the estimated maternal mortality ratio during the seven-year period prior to the survey was 576 maternal deaths per 100,000 live births while infant mortality rate was 69 deaths per 1,000 live births.[4] The under-five mortality rate was 37 per 1000 live births while the neonatal mortality rate was 31 per 1000 births.[4] Approximately 61% of pregnant women have at least one antenatal clinic attendance in Nigeria and only 38% of the deliveries are carried out by skilled birth attendants.[4] A significant proportion of mothers in developing countries still deliver at home unattended to by skilled birth attendants.[5] Individual factors that influence choice of birth place include maternal age, parity, education, marital status, household factors including family size and household wealth.[6] Others are community factors such as socioeconomic status, community health infrastructure, region, rural/urban residence, availability of the health facilities and distance to such health facilities.[7]

To achieve the sustainable Development Goal 3, it is required that at least 80% of all deliveries should take place in a health facility that has skilled birth attendant.[8] To enhance the utilization of health facilities during delivery in the country, barriers/determinants for utilizing such facilities during antenatal care and delivery among women need to be identified across all geographical regions.[9],[10] Previous studies have shown that sub-optimal care caused by lack of medical personnel, medical equipment, unavailability of drugs, high patient load and delay in attending to patients in the health facility, cultural beliefs and the influence of decision makers are some of the reasons patients choose to be delivered by the traditional birth attendants.[10],[11],[12],[13],[14],[15],[16] Other factors which encourage home delivery are poverty, aversion to caesarean section, distance and means of transport to the health facility and separation of the patients in labour from their family members at the health facility. [12] [13],[14],[15],[16]

Complications arising from pregnancy, delivery and postpartum period can be difficult to predict. In developing effective strategies for increasing the utilization of health facility delivery, it is necessary to understand the factors affecting choice of birth place. The underlying cause of low health facility-based delivery especially in rural areas needs further investigation and exploration in order to be better understood by the reproductive health planners. This informed the need to determine and analyze the factors that influenced pregnant women’s choice of place of delivery at Mater Misericordiae Hospital, Afikpo and Saint Vincent Hospital, Ndubia, two rural mission hospitals in Ebonyi State, Nigeria. This study was aimed at determining the factors that influence choice of birth place among antenatal clinic attendees at rural Mission hospitals in Ebonyi State. The findings from this study will help reproductive health planners and other stake-holders formulate policies aimed at enhancing the utilization of health facilities by pregnant women thereby reducing maternal morbidity and mortality.

## Materials and methods

### Study sites

Mater Misericordiae Hospital, Afikpo was founded in 1946 by the St Patrick Missionaries, a Roman Catholic based religious group. It is a secondary hospital that serves Afikpo community, the neighboring communities in Ebonyi and other surrounding States of Abia, Akwa Ibom, Cross River, Enugu and Imo. Majority of the patients treated in this hospital are low income earners and rural dwellers. Saint Vincent’s Hospital, Ndubia is also a secondary hospital located in Izzi Local Government Area of Ebonyi State. The hospital was established in the early 1960 by the Catholic missionaries. The hospital serves the rural population in Izzi, Ikwo, Ezza and neighbouring states of Cross River and Benue. Most of the patients are rural dwellers and are predominantly farmers.

### Study design

This was a cross-sectional study in which interviewer-administered semi-structured questionnaires were used to extract information from the consenting antenatal clinic attendees who had history of previous delivery prior to the index pregnancy. The questionnaires were administered by the researchers and trained research assistants. Prior to this study, the questionnaires were pre-tested on 20 antenatal clinic attendees at the Federal Teaching Hospital, Abakaliki.

Each of the questionnaires had six sections and these sections comprised the participants’ socio-demographic characteristics, accessibility to healthcare services, previous antenatal and delivery history, rating of public and private health facilities, the pregnancy risks and recommendation for good maternal and perinatal outcome. The number of study participants recruited from each centre was based on the proportion of antenatal clinic attendees in each of the study centres. The study participants were consecutively recruited until the number allocated to each centre was completed.

The eligible participants in this study were antenatal clinic attendees who had history of delivery within 3 years prior to the index pregnancy and those who gave consent to participate in the study. However, the exclusion criteria comprised participants whose questionnaires were incompletely filled, nulliparous women and women who, despite adequate counseling, declined to participate in the study.

The minimum sample size for the study was calculated using the sample estimation formula for cross sectional studies,[17]

$$\text{n}=\frac{\text{p}\left(\text{1-p}\right)\;{\left(\text{Z}\right)}^{\text{2}}}{{\text{e}}^{\text{2}}}$$

P = prevalence of delivery in Nigerian health facilities = 38%[4]; 1-p = number of deliveries outside established health facility = 1–0.38 = 0.62

e = standard error = 5% and Z = standard normal variance = 1.96 at

95% confidence interval.

Adding a 10% attrition rate, n was 398

Data were analyzed with EPI Info 7.21 software (CDC Atlanta Georgia).

The ethical clearance for this study was obtained from the Ethics Committee of the Federal Teaching Hospital, Abakaliki. Institutional Permission was also obtained from Mater Misericordiae Hospital, Afikpo and St. Vincent Hospital, Ndubia before the commencement of this study. Confidentiality was achieved on all the study participants through the use of anonymous questionnaires and the information was individually obtained from each of the participants.

## Results

A total of 250(62.5%) and 150(37.5%) questionnaires were distributed at Mater Misericordiae Hospital, Afikpo and Saint Vincent’s Hospital, Ndubia respectively. However,397(99.3%) questionnaires were completely filled. Therefore the information from 397 questionnaires was collated and analyzed. Table 1 shows the socio-demographic characteristics of the participants. Majority of the participants were between 25 and 34 years, christians, grand multiparous, married, Igbos, farmers and had primary level of education. The mean age of the eligible participants was 28.67±3.4 years.

**Table 1:** The socio-demographic characteristics of the respondents.

Age	Frequency	Percentage
15–24	87	21. 92
25–34	256	64.48
35–44	54	13.60
**Religion**		
Christianity	347	87.41
Islam	20	5.04
African traditional religion	30	7.55
**Parity**		
1	100	25.19
2–4	138	34.76
≥ 5	159	40.05
**Ethnicity**		
Igbo	357	89.92
Hausa	14	3.53
*Others	26	6.55
**Marital status**		
Married	304	76.57
Single	34	8.56
Windowed	33	8.32
Divorced/separated	26	6.55
**Level of education**		
No formal education	76	19.14
Primary	149	37.53
Secondary	104	26.20
Tertiary	68	17.13
**Husband educational level**		
No formal education	92	23.17
Primary	52	13.10
Secondary	176	44.33
Tertiary	77	19.40
**Occupation of the respondent**		
House wife	76	19.14
Farming	169	42.57
trading	90	22.67
Civil/public service	62	15.62
**Husband occupation**		
Farming	172	43.32
Trading	117	29.47
Civil/public service	108	27.21

Most of the respondents’ husbands had secondary level of education and were farmers. Table 2 shows the respondents’ accessibility to healthcare services. It took majority of the study participants more than 5 hours to access the healthcare facilities and most of the healthcare facilities were privately owned. Though 71% of the health facilities closest to the respondents had maternity services, 29% of such facilities did not have such services. It took at least 1 hour for 81% of the respondents to access health facilities with maternity services and majority (55%)of them access such facilities by vehicles and motor cycles. The respondents’ previous antenatal history is shown in Table 3. Most (60.2%) of the respondents had antenatal care attendance in their previous pregnancy and a significant proportion of the previous ANC attendees did so at public hospitals and for more than 4 times. Table 4 shows the factors that influenced the respondents’ birth place at the last delivery. Majority (43.8%) of the respondents were delivered by the skilled birth attendants in their last delivery. The most common determinant for the choice of birth place by the respondents was nearness of the health facility to the respondents. Approximately 57.2%) of the respondents did not have transport fare to take them to health facility with maternity services. Majority (42.8%) of the respondents took the decision on birth place themselves. The influence of socio-demographic characteristics on delivery by skilled birth attendants among the respondents is shown in Table 5. The respondents’ age and educational level, their husbands’ educational level, history of antenatal clinic attendance, distance of the health facility and availability of transport fare had a significant effect on delivery by skilled birth attendants. Table 6 shows the respondents’ choice of birthplace at the index pregnancy. Most (61.7%) of the respondents chose to deliver in public health facilities and the main reasons for such choices were cost, better services and familiarity of the health workers to the respondents. Table 7 shows the respondents’ rating of services in the health facilities. The respondents answered that majority of health workers in public health facilities rendered good services, were skilled, had good infrastructure and enough drugs. However a significant proportion of the public health workers were said to be rude to patients. The participants also responded that majority of the health workers in private health facilities were caring, render good services and had good quality of infrastructure but had shortage of drugs. However, the participants responded that a significant proportion of private health workers were unskilled.

**Table 2:** Respondents’ accessibility to healthcare services.

**Time it took to reach the nearest health facility from home**	**Frequency (N = 397)**	**Percentage**
Close (<1hour)	109	27.46
Far (1-4hours)	99	24.94
Very far (≥5hour)	189	47.60
**Type of nearest health facility**		
Public	177	44.58
Private	220	55.42
**Availability of maternity services in nearest health facility**		
Yes	282	71.03
No	115	28.97
**Closest maternity services where nearest health facility does not have maternity services.**	**N = 115**	
Close (<1hour)	22	19.13
Far (1-4hours)	58	50.43
Very far (≥5hours)	35	30.44
**Means of transportation to health facility with maternity services**		
Foot	136	34.26
Bicycle	44	11.08
Motorcycle	90	22.67
Vehicle	127	31.99

**Table 3:** The respondents’ previous antenatal history.

**Attendance to antenatal clinic in previous pregnancy**	**N = 397**	**Percentage**
Yes	239	60.20
No	158	39.80
**Place of antenatal care**	**N = 239**	
Public only	172	71.97
Private only	38	15.90
Both private and public	29	12.13
**Frequency of antenatal**	N = 239	
1–2	36	15.06
3–4	52	21.76
>4	151	63.18

**Table 4:** Factors that influenced the respondents’ birth place at the last delivery.

**Birth place at last delivery**		
Home	78	19.65
TBA	145	36.52
Public health facility	134	33.75
Private health facility	40	10.08
**Determinants of birth place**		
Nearness	97	24.43
Familiarity of health providers to the respondents	90	22.67
Cost	38	9.57
Better service	97	24.43
Sudden onset of labour	75	18.89
**Distance (Length of time in reaching health facility with maternity services)**		
Close (< 1 hour)	84	21.16
Far (1-< 3 hours)	138	34.76
Very far (≥3hours)	175	44.08
**Means of transportation**		
Foot	124	31.23
Bicycle	41	10.33
Motorcycle	90	22.67
Vehicle	140	35.27
**Availability of transport fare**		
Yes	170	42.82
No	227	57.18
**Who determined place of delivery**		
Self	170	42.82
Husband	110	27.71
Mother	46	11.59
Health worker	31	7.80
Found self-there (complications)	40	10.08

**Table 5:** The influence of socio-demographic characteristics on the respondents’ delivery by the skilled birth attendants.

Socio-demographc characteristics	Delivered by the skilled birth attendants	Not delivered by the skilled birth attendants	X^2^	P-value
Age (Years)			16.538	0.0001*
≤24	21	66		
≥25	153	152		
Parity			2.175	0.1403
1	21	63		
≥2	153	160		
Educational level			20.378	0.0001*
≤Primary	76	149		
≥Secondary	98	74		
Husbands’ educational level			4.091	0.0431*
≤Primary	53	91		
≥Secondary	121	132		
Attendance to antenatal clinic			70.909	0.0001*
Yes	146	93		
No	28	130		
Distance (time it took to reach the health facility).			8.373	0.0038*
<1 hour	49	35		
≥ 1 hour	125	188		
Availability of transport fare			28.229	0.0001*
Yes	101	69		
No	73	154		

* = Statistically significant.

**Table 6:** The respondents’ choice of birthplace in the index pregnancy.

**Choice of birthplace**	Frequency (N = 397)	%
Traditional birth attendant	41	10.33
Public health facility	245	61.71
Private health facility	111	27.96
**Main reason for choice of birth place**		
Nearness	66	16.62
Familiar faces	126	31.74
Cost	98	24.69
Better services	107	26.95
**Choice of birth place if all factors are favourable**		
TBA	46	11.49
Public health facility	194	48.87
Private health facility	157	39.54

**Table 7:** The respondents’ rating of services in the health facilities.

Input	Indicator	Public (%) N = 397	Private (%) N = 397
*Health workers attitude	Rudeness	269(46.30)	78(13.43)
	Caring	185(31.84)	350(60.24)
	Sympathetic	127(21.85)	153(26.33)
Skills	Skilled	321(80.86)	151(38.04)
	Unskilled	76(19.14)	246(61.96)
Quality of services	Excellent	88(22.17)	38(9.57)
	Good	175(44.08)	200(50.38)
	Fair	101(25.44)	22(5.54)
	Poor	33(8.31)	137(34.51)
Quality of infrastructure	Excellent	116(29.22)	53(13.35)
	Good	174(43.83)	181(45.59)
	Fair	79(19.90)	122(30.73)
	Poor	28(7.05)	41(10.33)

*multiple answers were allowed.

## Discussion

This study showed that there was a distant location of the healthcare facilities to most of the respondents in their previous deliveries. Majority of such facilities were privately owned. Though 60.2% of the respondents attended antenatal care clinics in their previous pregnancies, It was only 43.8% of them that were delivered by skilled birth attendants. The most common determinant for the choice of birth place by the respondents was nearness of the health facility to the respondents. It took at least 1 hour for 81%% of the respondents to reach health facility with maternity services and majority of them usually reached the health facility by vehicles and motor cycles. However most of the respondents could hardly afford the transport fare to take them to such facilities. The age of the respondents and their husbands, their educational qualification, history of antenatal clinic attendance, location of the health facility and availability of transport fare had a significant effect on delivery by the skilled birth attendants. Most of the respondents chose to deliver in public health facilities in the index pregnancies.

The distant location of health facilities with maternity services which was considered a major constraint by the respondents in this study is a common problem in resource-poor settings. This is supported by previous studies in sub-Saharan Africa and Asia.[9],[10],[12],[16] With only 60.2% of the respondents who had ever attended an antenatal care clinic in their last pregnancy, there is an under-utilization of antenatal care services in this environment. This is essentially similar to the 61% of Nigerian pregnant women who had at least one antenatal clinic attendance but much lower than 94% reported in Zambia.[4],[18]

The 56.17% of deliveries carried out by unskilled personnel in this study is slightly lower than the Nigeria national figure of 62% but higher than the reports from the Bahi District, central Tanzania, Eastern rural Nepal and Mukona District of Uganda [4],[9],[12],[14] This poses a public health challenge as in spite of 60.2% of at least one antenatal clinic attendance in the preceding pregnancy of the respondents, it was only 43.8% of the antenatal clinic attendees that delivered in a health facility. The 43.8% of the respondents delivered by skilled birth attendants in this study is marginally higher than the Nigerian national figure of 38%.[4] This is worrisome as no significant progress has been made towards improving the proportion of pregnant women delivered by the skilled birth attendants in this environment. The significant influence of age, educational level of the respondents and their spouses, history of antenatal clinic attendance, distance of the health facility from the respondents and availability of transport fare on delivery by skilled birth attendants in this study is essentially similar to previous reports in Ghana and Kenya.[19],[20] Some of the reasons given by respondents for the choice of place of delivery like nearness of the health facility to the respondents, familiarity of the health workers to the respondents, cost of the delivery, improved services, sudden onset of labour, means of transportation and the person who takes decision on the delivery site are essentially similar to the previous reports in Malawi and Uganda.[13],[14]

The study showed that more than half of the respondents would prefer to deliver in a public health facility in this index Pregnancy. The factors that influenced their choice of public health facilities were nearness of the public health facilities to the women, familiarity of health workers to the patients, skilled man power, cheaper cost of delivery and improved services. However, rudeness of health workers in public health facilities could be an inhibitory factor that may prevent patients from seeking delivery in such facilities. A significant proportion of the respondents would also like to deliver in private health facilities if not because of high cost of delivery services and lack of skilled personnel and drugs at such facilities. The perceived difference in the quality of care offered by the public and private health facilities in this study is similar to the previous reports in Mukono District, Uganda, Kano, Nigeria and rural Orissa, India respectively.[14],[17],[21] The perception among the respondents that health workers in public health facilities were more skilled than those in private health facilities in this study is similar to the previous studies in Uganda, Kano and India respectively.[14],[21],[22] While it was noticed that health workers in the public health facilities were rude, those in the private facilities were more caring and sympathetic and this was essentially similar to previous reports. [14],[23],[24] Rudeness of health workers to pregnant women at a health facility can further reduce their antenatal clinic attendance and delivery by skilled birth attendants at such facility. To address this barrier, the health workers need to be trained on Respectful Maternity Care.[25] Contrary to previous reports in Kano, Nigeria,[14] drugs were more available in public health facilities than private facilities in this study.

### Limitations

This study was limited by the information provided by the respondents which was prone to recall bias. It was also a hospital-based study in which its findings may not be a true reflection in the society. It was further weakened by the recruitment of the study participants at the antenatal clinic when most pregnant women who delivered at home might not have attended such clinic. This could have been responsible for the marginal increase in delivery by skilled birth attendants above the Nigerian national average in this study.

## Conclusion

This study has shown that greater proportion of pregnant women will prefer to deliver in a health facility. However there are barriers militating against the utilization of these facilities for delivery in spite of a significant proportion of the women having attended antenatal clinic. This underscores the need for education and socio-economic empowerment of the couple and the health policy makers should design programmes aimed at ensuring free antenatal care. The Federal and State ministries of Health in Nigeria should carry out effective monitoring of health facilities in order to ensure that such facilities maintain at least a minimum standard before they are allowed to operate. There is also need for training and re-training of health workers on Respectful Maternity Care which will help reduce their rudeness and other violent practices to patients. Government and other stakeholders may provide incentives as one of the ways of enhancing proper utilization of the healthy facilities by these women to ensure safe delivery.

## Supporting information

S1 File Questionnaire for choice of birth place. (DOCX)
