# Supplementary material for: Choice of birth place among antenatal clinic attendees in rural mission hospitals in Ebonyi State, South-East Nigeria
Source: PLoS One. 2019 Nov 5;14(11):e0211306. doi: 10.1371/journal.pone.0211306 (PMC6830769; doi:10.1371/journal.pone.0211306)
Supplement: S1 File — (DOCX) [file pone.0211306.s001.docx]

**QUESTIONNAIRE FOR CHOICE OF BIRTH PLACE**

**QUESTIONNAIRE Date of Interview: __________**

**SECTION A: DEMOGRAPHIC INFORMATION**

1. Address:_____ 2.Religion _________ 3.Denomination __________

4.Age_________5.Parity:_________6.Tribe:________________

**TICK YOUR CHOSEN OPTIONS IN THE BOXES FROM 7 BELOW:**

7. Marital status a. Single [ ] b. Married [ ] c. Separated [ ] d. Widowed [ ]

8. Level of Education a. None [ ] b. primary [ ] c. Secondary [ ] d. Tertiary [ ]

9. Education of Husband a. None [ ] b. primary [ ] c. Secondary [ ] d. Tertiary [ ]

10. Occupation of respondent a. None [ ] b. farming [ ] c.Trading [ ]

d. Formally employed [ ]

11. Occupation of husband a. None [ ] b. farming [ ] c.Trading d. formally employed [ ]

**SECTION B: ACCESSIBILITY INFORMATION TO HEALTH SERVICES**

12. How far is the nearest health facility from your home?

a. close(<1hour) [ ] b. far(1-4hours) [ ] c. very far (>5hours)[ ]

13. What type of health facility is it? a. Public [ ] b. Private [ ]

14. Has it any maternity services? a. Yes [ ] b. No [ ]

15. If no, how far is the nearest health facility that offers maternity services?

a. close(<1hour) [ ] b. far(1-4hours) [ ] c. very far(>5hours) [ ]

16. What means of transport do you use to reach the health facility?

a. On foot [ ] b. Motorcycle [ ] c. Bicycle [ ] d. vehicle [ ]

**SECTION C: ANTENATAL INFORMATION**

17. Did you attend antenatal clinic during your previous pregnancy?

a. Yes [ ] b. No [ ]

18. If yes, where did you attend Antenatal care? a. Traditional birth attendant [ ]

b. Public Clinic [ ] c. Private clinic [ ] d. both private and Public clinics[ ] e. None

19. How many times did you visit the clinic? a. Once [ ] b. two [ ] c. Thrice [ ] d. four [ ] e. Over 4 times [ ] f. None [ ]

**SECTION D: DELIVERY INFORMATION**

20. Where did you deliver during your previous pregnancy? a. At home [ ]

b. By a traditional birth attendant [ ] c. Public Clinic [ ] d. Private Clinic [ ]

21. Why did you choose to deliver there? a. nearness [ ] b. familiarity of the health workers [ ] c. cost [ ] d. labour was sudden [ ]

22. How far was it from your home?

a. close(<1hour) [ ] b. far(1-4hours) [ ] very far(>5hours) [ ]

23. How long did it take you to reach the facility when labour started?

a. Less than 1 hour [ ] b. 1-2 hours [ ] c. 3 – 4 hours [ ] d. ≥ 5 hours [ ]

24. What means of transport did you use?

a. Foot [ ] b. Motorcycle [ ] c. Bicycle [ ] d. Vehicle [ ]

25. Did you have money for transport when labour started?

a. Yes [ ] b. No [ ]

26. Who determined the site where you delivered your baby? a. Self [ ] b. Health workers[ ] c. Husband [ ] d. Other relatives [ ]

**SECTION E: RATING PUBLIC AND PRIVATE CLINICS**

27. Where would you want to deliver in index pregnancy?

a. At home b. By the traditional birth attendant [ ] c. private hospital [ ] d. public hospital [ ]

28. If all factors were favourable, where would you have preferred to deliver?

a. At home [ ] b. Private hospital [ ] c. By the traditional birth attendant [ ]

d. Public hospital [ ]

29. Why? a. Distance [ ] b. Hospitality [ ] c. Costs [ ] e. Good infrastructure [ ]

30. How do you rate the health workers in private hospitals?

a. Rude [ ] b. Caring [ ] c. Sympathetic [ ]

31. How do you rate health workers in Public hospitals?

a. Rude [ ] b. Caring [ ] c. Sympathetic [ ]

32. How do you rate the services in Public hospitals?

a. Excellent [ ] b. Fair [ ] c. Good [ ] d. Poor [ ]

33. How do you rate the services in Private hospitals?

a. Excellent [ ] b. Fair [ ] c. Good [ ] d. Poor [ ]

34. How do you rate the infrastructure in Public hospitals?

a. Excellent [ ] b. Fair [ ] c. Good [ ] d. Poor [ ]

35. How do you rate the infrastructure in Private hospitals?

a. Excellent [ ] b. Fair [ ] c. Good [ ] d. Poor [ ]

36. How do you rate the skill of health personal in the public sector

a. skilled [ ] b. unskilled [ ]

37. How do you rate the skill of health personal in the private sector

a. skilled [ ] b. unskilled [ ]

36. Are supplies and medicines always available in Public hospitals?

a. Yes [ ] b. No [ ]

37. Are supplies and medicines always available in Private hospitals?

a. Yes [ ] b. No [ ]

**THANK YOU MADAM FOR YOUR TIME AND CO-OPERATION.**
